# Non‐Carious Cervical Lesions in Wild Primates: Implications for Understanding Toothpick Grooves and Abfraction Lesions

**DOI:** 10.1002/ajpa.70132

**Published:** 2025-09-29

**Authors:** Ian Towle, Kristin L. Krueger, Kazuha Hirata, Mugino O. Kubo, Anderson T. Hara, Joel D. Irish, Carolina Loch, Matthew R. Borths, Luca Fiorenza

**Affiliations:** ^1^ Biomedicine Discovery Institute, Department of Anatomy and Developmental Biology Monash University Melbourne Victoria Australia; ^2^ Department of Anthropology Loyola University Chicago Chicago Illinois USA; ^3^ Graduate School of Science Kyoto University Inuyama Aichi Japan; ^4^ Department of Natural Environmental Studies, Graduate School of Frontier Sciences The University of Tokyo Kashiwa Chiba Japan; ^5^ Oral Health Research Institute, Department of Biomedical and Applied Sciences Indiana University School of Dentistry Indianapolis Indiana USA; ^6^ School of Biological and Environmental Sciences Liverpool John Moores University Liverpool UK; ^7^ Centre for the Exploration of the Deep Human Journey University of the Witwatersrand Witwatersrand South Africa; ^8^ Sir John Walsh Research Institute, Faculty of Dentistry University of Otago Dunedin New Zealand; ^9^ Duke Lemur Center Museum of Natural History Duke University Durham North Carolina USA

**Keywords:** fossil hominins, *Homo* diet, interproximal grooves, NCCLs, root lesions

## Abstract

**Objectives:**

In clinical settings, non‐carious cervical lesions (NCCLs) are often linked to abrasion, erosion, abfraction, or a combination of these factors. In archaeological and paleontological remains, the most common NCCL is the “toothpick groove,” yet little is known about the occurrence of these and other NCCLs in wild non‐human primates.

**Materials and Methods:**

Here, we examine 531 individuals from 27 wild extant and extinct anthropoid primate taxa for NCCLs. Macroscopic examinations were followed by microwear and tissue‐loss analyses using multiple imaging techniques, including stereoscopic microscopy, confocal laser, handheld digital microscopy, and 3D tissue loss analysis.

**Results:**

NCCLs were identified in 21 individuals, indicating a prevalence of 4% within the sample. The distribution of NCCLs was uneven, with multiple cases concentrated in certain taxa and populations, but they are identified in all major groupings (e.g., Platyrrhini, Cercopithecidae, Hominoidea). Two distinct lesion types were identified: (1) localized U‐shaped lesions with internal parallel striations, indicative of regular contact with abrasive materials (i.e., attrition or abrasion); and (2) smooth, shallow lesions characterized by tissue loss along the recessed gum line, indicative of a multifactorial process that may involve acid erosion.

**Discussion:**

Several attrition/abrasion NCCLs resembled or have characteristic features of “toothpick grooves” known from hominin samples, suggesting the need for further comparative analyses between human and non‐human primates. The absence of abfraction lesions supports the view that abfraction may be related to contemporary human behaviors. These findings emphasize the value of non‐human primate data for interpreting NCCLs in both contemporary and ancient human populations.

## 1 Introduction

Non‐carious cervical lesions (NCCLs) are common in contemporary clinical contexts (e.g., Teixeira et al. 2020; Kitasako et al. 2021). Extensive research over the past few decades has explored their complex etiology, progression, and restorative challenges. Recent studies suggest NCCLs often arise from the interplay of mechanical and chemical factors (Alvarez‐Arenal et al. 2019; Villamayor et al. 2024; Denucci, Towle, et al. 2024). Specifically, both clinical observations and experimental models show that NCCLs result from a multifactorial contribution of abrasion (item‐on‐tooth contact; e.g., improper tooth brushing), attrition (tooth‐on‐tooth contact; e.g., malocclusion), erosion (tooth‐on‐acid contact; e.g., exposure to dietary or intrinsic acids), and abfraction (some researchers consider occlusal loading leads to cervical microfractures; Lee and Eakle 1984; Grippo 1991; Rees 2002). Recent research has also highlighted the role of masticatory forces in initiating NCCLs (Alvarez‐Arenal et al. 2019; Dioguardi et al. 2024). While certain surfaces are more commonly affected, and lesion morphology may suggest a likely cause (Igarashi et al. 2017; Goodacre et al. 2023), the shape of a lesion alone may not be a reliable predictor of its etiology (Pecie et al. 2011).

In contrast, NCCLs in archaeological and paleontological contexts typically show a different pattern. Abfraction (wedged‐shaped lesions) is absent and erosion is rarely reported (Ritter et al. 2009; Urzúa et al. 2015; Towle et al. 2018). One type of NCCL, the “toothpick grooves,” or interproximal grooves, has been frequently reported in the literature. These grooves have been studied for over a century (e.g., Siffre 1911; Martin 1923; Campbell 1925), and are documented across a wide range of archaeological samples and different *Homo* species (e.g., Campbell 1925; Frayer and Russell 1987; Brown and Molnar 1990; Fong 1991; Durband et al. 2012; Sun et al. 2014; Estalrrich et al. 2017; Nowaczewska et al. 2021; Neves et al. 2022). While these lesions are most commonly reported on posterior permanent teeth, they have also been reported on anterior and deciduous teeth (Schulz 1977; Frayer and Russell 1987; Ricci et al. 2016; Willman et al. 2019).

Several hypotheses have been proposed regarding the origins of these interproximal NCCLs in archaeological and paleontological dental samples, including localized gingivitis (Weidenreich 1937), dental erosion (Campbell 1925; Brothwell 1963; Pindborg 1970), and grit abrasion along exposed root surfaces (Wallace 1974). However, the prevailing view is that these lesions result from the repeated, deliberate use of objects such as plant fibers, wood, or bone to remove food debris or alleviate dental discomfort (Ubelaker et al. 1969; Berryman et al. 1979; Frayer and Russell 1987; Formicola 1988; Hlusko 2003; Estalrrich et al. 2017; Frayer et al. 2017; Willman et al. 2019). Experimental studies have shown that such implements can produce grooves consistent with NCCLs observed in fossil hominins, with some suggesting that the incorporation of grit during tooth picking may play an important role in lesion formation (Berryman et al. 1979; Teaford and Walker 1983; Bermudez de Castro et al. 1997; Hlusko 2003).

The examination of NCCLs in non‐human primates can offer important insights into the potential evolutionary and biomechanical factors underlying lesion formation. Given their close phylogenetic relationship to humans, extant and extinct non‐human anthropoid primates (Platyrrhini and Catarrhini) share many physiological, dental, and behavioral traits that can inform the study of NCCLs. This study examines the prevalence of NCCLs among anthropoid primates and provides a detailed morphological and microscopic analysis and description of the observed lesions. Our goal is to determine whether NCCLs commonly observed in clinical and fossil *Homo* samples also occur in our closest primate relatives. Here, we make two hypotheses: First, lesions often associated with erosion and abrasion in clinical contexts will occur in the sample (erosion: shallow, smooth lesions on exposed root surfaces; abrasion/attrition: U‐shaped lesions with clear, parallel striations), reflecting the acidic fruit diets common among some primate taxa (Milton 1993; Valenta et al. 2020) and the mastication of hard items observed in others (Towle, MacIntosh, et al. 2022). Second, abfraction lesions, characterized by deep wedged or V‐shaped lesions, and interproximal toothpick grooves, which are confined to the interproximal root surfaces and feature tapered, shallow profiles with fine parallel striations and considered unique to *Homo*, will not be present in non‐human primates.

## 2 Materials and Methods

The specimens studied are curated at five institutions: Powell‐Cotton Museum (United Kingdom; Institution Code PCM); Primate Research Institute, Kyoto University (Japan; Institution Code PRI; now the Center for the Evolutionary Origins of Human Behavior); Duke Lemur Center Museum of Natural History at Duke University (USA; Institution Code DPC); Field Museum of Natural History in Chicago (USA; Field Museum; Institution Code FMNH); and the Natural History Museum in London (United Kingdom; Institution Code NHMUK). In total, 531 individuals from 27 extant and extinct anthropoid taxa were studied. The data that supports the findings of this study, including a complete list of specimens, including species identification, geographic origin, and curation details, is available in the Supporting Information. All specimens were obtained from wild populations; however, Japanese macaques ( *Macaca fuscata* ) from Koshima Island (Japan) were regularly provisioned as part of a primate field study (Kawai 1963; Mori 1995; Watanabe 1989). For isolated teeth from *Kenyapithecus* and *Griphopithecus*, we did not attempt to differentiate or assign to species level (see Kelley et al. 2008 for a taxonomic review of these specimens).

NCCLs were identified by the presence of macroscopically visible tissue loss on the cervical aspect of the tooth, not associated with carious tissue loss, with the latter identified by clear cavitation associated with demineralization and a rough textured lesion (see below; Towle, Irish, et al. 2022). Lesions were documented using a digital camera and a Dino‐Lite handheld microscope (Dino‐Lite AM2111). For specimens from the Field Museum and the PRI, microwear analysis was conducted using different imaging modalities: a stereoscopic microscope (Olympus SZX10) for Field Museum specimens and a confocal laser microscope (VK‐9700, Keyence, Osaka, Japan) for PRI specimens.

In this study, differential diagnosis between caries and NCCLs was undertaken. Root carious lesions result from demineralization, often extending deep into the dentine, and are characterized by a rough and/or softened texture, changes in localized coloration, and an irregular morphology distinct from tissue loss caused by tooth wear. Active lesions often appear dark, with poorly defined borders or undermining of the tooth structure, and are typically asymmetrical or non‐uniform in shape, texture, and coloration. In contrast, NCCLs often lack intrinsic discoloration, often appear shiny, and present as well‐demarcated, uniform/symmetrical lesions with no evidence of demineralization (e.g., no visible rough texture or soft texture under probing). In osteological and fossil material, diagnosis can be more challenging due to taphonomic alteration. For this study, we excluded lesions that were clearly carious based on these criteria and only included those consistent with NCCLs. However, where lesions displayed ambiguous features, or multiple potential lesion types were visible on the same tooth surface, specimens were included and discussed in detail.

Standard molding procedures were used for both PRI and Field Museum samples, following established protocols (Field Museum samples: Krueger 2015; Krueger et al. 2019; PRI samples: Kubo and Kubo 2014; Kubo et al. 2017; Kubo et al. 2021; Towle, MacIntosh, et al. 2022). Images were captured at magnifications ranging from 10× to 100× to assess NCCL characteristics such as size, orientation, and striation patterns. Specimens from the Field Museum were also scanned in high resolution using an intraoral 3D scanner (3Shape Trios 4; Amornvit et al. 2021). Each specimen with one or more NCCL was compared with clinical, archaeological, and paleontological literature.

A specific case study of an orangutan ( *Pongo pygmaeus* ) specimen, FMNH 19026, underwent further analysis. Tissue loss was assessed using a 3D mesh generated by the intraoral scanner. The original root morphology was reconstructed by referencing the antimere and unaffected areas of the tooth using Meshmixer (Autodesk Inc., San Rafael, CA, USA). To do this, we utilized “Bridge,” “Hole Fill,” and various sculpt “Brushes” to approximate the tooth root's appearance prior to NCCL formation. The carious lesions beneath the NCCL were left unaltered. The volumes of the original and “NCCL‐filled” 3D meshes were calculated using Meshlab (Cignoni et al. 2008), with the difference providing an estimate of the dental tissue lost during NCCL formation. Additionally, *WearCompare* (LeedsDigitalDentistry, UK) was used to visualize and quantify tissue loss by superimposing two 3D meshes (O'Toole et al. 2020; Towle, Krueger, et al. 2024). The data that support the findings of this study are available in the Supporting Information.

## 3 Results

Among the 531 anthropoid specimens studied, 21 individuals (4% of the sample) displayed one or more NCCLs. The prevalence of NCCLs varied markedly among different groups (Table 1). This analysis includes NCCLs that share characteristics with toothpick grooves observed in archaeological and paleontological contexts. However, no lesions consistent with abfraction (i.e., wedged or V‐shaped lesions that penetrate deep into the cervical dentine), commonly present in clinical settings, were observed.

**TABLE 1:** Summary of species studied, total individuals, and NCCL occurrence.

Taxa	Common name/Fossil age	Total *n* individuals	Individuals with NCCLs (%)
*Pan troglodytes*	Chimpanzee	109	0 (0)
*Gorilla gorilla gorilla*	Western lowland gorilla	83	2 (2.4)
*Pongo pygmaeus*	Bornean orangutan	9	2 (22.2)
*Hylobates klossii*	Kloss's gibbon	15	0 (0)
*Theropithecus gelada*	Gelada	8	0 (0)
*Papio hamadryas*	Hamadryas baboon	20	0 (0)
*Mandrillus* spp.	Mandrill	10	0 (0)
*Lophocebus albigena johnstoni*	Johnston's mangabey	8	2 (25)
*Macaca fuscata*	Japanese macaque	48	9 (18.8)
*Cercopithecus denti*	Dent's mona monkey	10	1 (10)
*Cercopithecus mitis*	Blue monkey	8	0 (0)
*Piliocolobus oustaleti*	Oustalet's red colobus	7	0 (0)
*Presbytis femoralis*	Raffles' banded langur	8	3 (37.5)
*Presbytis potenziani*	Mentawai langur	8	0 (0)
*Simias concolor*	Pig‐tailed langur	20	0 (0)
*Lagothrix lagothricha*	Brown woolly monkey	7	1 (14.3)
*Pithecia* spp.	Saki	8	0 (0)
*Alouatta seniculus seniculus*	Colombian red howler	10	0 (0)
*Sapajus apella*	Tufted capuchin	9	0 (0)
*Parapithecus grangeri*	Early Oligocene; ~29 Ma	16	0 (0)
*Catopithecus browni*	Late Eocene; ~34 Ma	11	0 (0)
*Propliopithecus* (*P. chirobates* and *P. ankeli*)	Early Oligocene; ~29 Ma	18	0 (0)
*Apidium phiomense*	Early Oligocene; ~29 Ma	19	0 (0)
*Aegyptopithecus zeuxis*	Early Oligocene; ~29 Ma	21	1 (4.8)
*Proconsul* ( *P. africanus* and *P. major* )	Early Miocene; ~19 Ma	5	0 (0)
*Ekembo nyanzae*	Early Miocene; ~19 Ma	3	0 (0)
*Kenyapithecus* and *Griphopithecus*	Middle Miocene; ~14 Ma	33	0 (0)

*Note:* For fossil specimens, an individual may be represented by only a few, or even a single tooth. Geological age for fossil specimens from Seiffert (2010) and Werdelin (2010).

### 3.1 U‐Shaped NCCLs With Parallel Striations Indicative of Abrasion/Attrition

**FIGURE 1:**
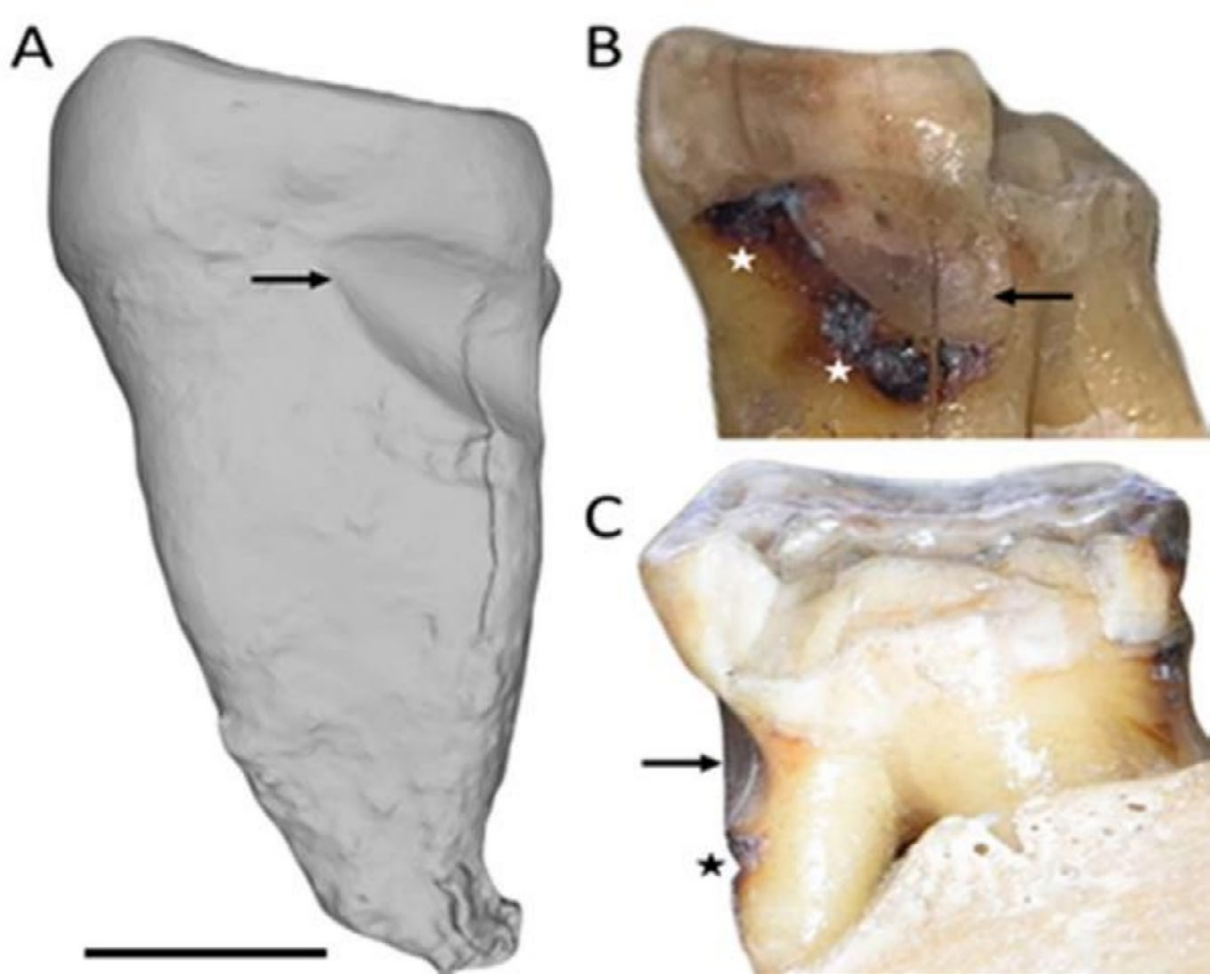
Lower left second molar of specimen FMNH 19026 ( *Pongo pygmaeus* ) showing a non‐carious cervical lesion (NCCL). (A) Overall view of the mesial surface of the crown and root; (B) Buccal/mesial view of the NCCL; (C) Buccal view of the NCCL. Arrows indicate the location of the NCCL, and stars mark dental caries. Scale bar: 5 mm.

**FIGURE 2:**
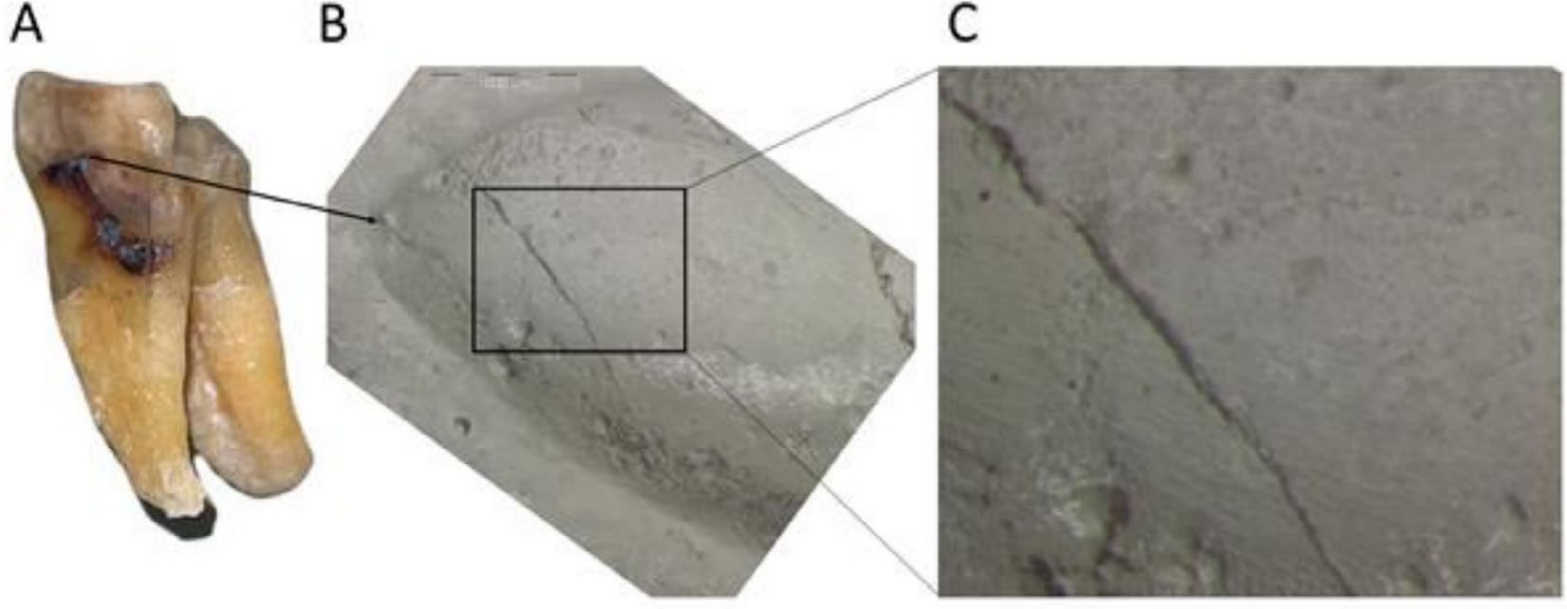
Microwear analysis of the non‐carious cervical lesion (NCCL) in Figure 1, showing the lower left second molar of FMNH 19026 ( *Pongo pygmaeus* ). (A) Overview of the tooth in mesial/buccal view; (B) Overview of the NCCL (20X magnification), scale 1000 μm. (C) High magnification view of the microwear (60X magnification), showing parallel buccal‐lingual fine striations within the NCCL.

The mesial surface of the lower left second molar of specimen FMNH 19026 ( *Pongo pygmaeus* ) showed a distinct NCCL resembling a toothpick groove, similar to those observed in archaeological and fossil *Homo* samples. The groove begins as a relatively wide indentation on the buccal side of the interproximal root surface and gradually tapers just past the midpoint of the interproximal surface (Figure 1A). The lesion follows the cementoenamel junction (CEJ) and an adjacent carious lesion located apical to the groove (Figure 1B,C). Despite the adjacent caries, the groove itself was non‐carious in nature, displaying a polished, smooth appearance macroscopically. Microwear analysis revealed parallel fine striations within the groove oriented buccal‐lingually (Figure 2A–C).

**FIGURE 3:**
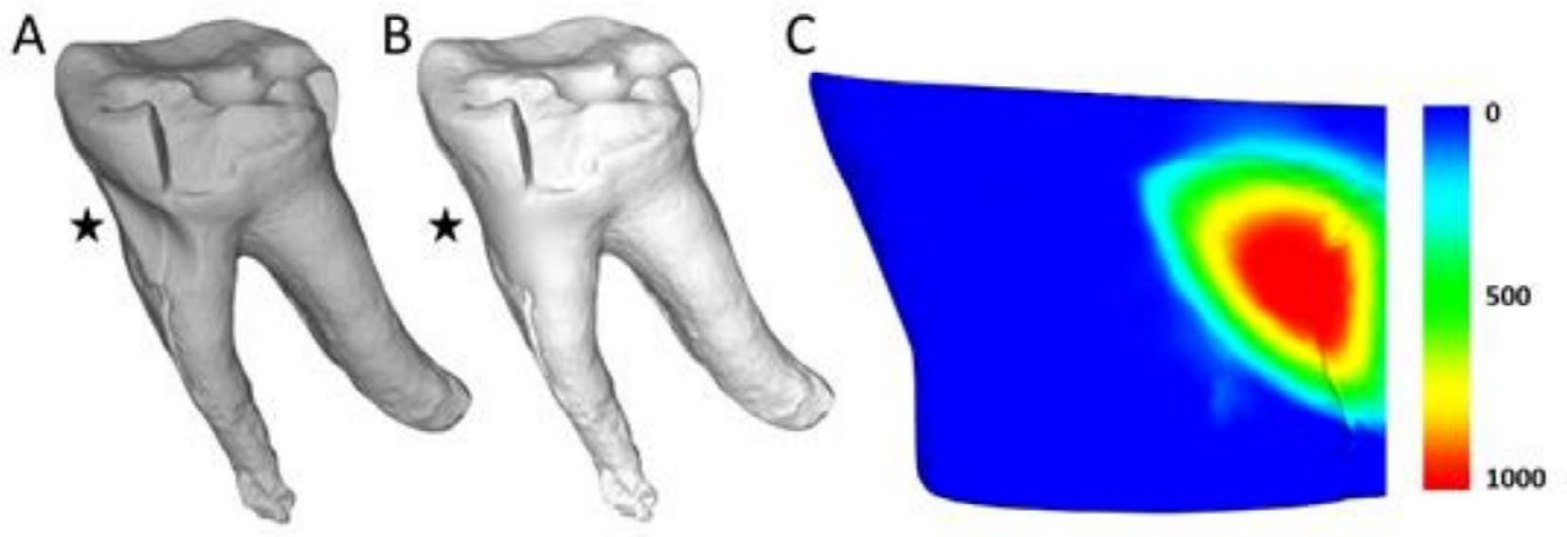
(A) Original 3D model of the lower left second molar of specimen FMNH 19026 ( *Pongo pygmaeus* ) NCCL; (B) Same 3D model but with the NCCL virtually filled to approximate the original root morphology. The star marks the location of the NCCL in both models; (C) Tissue loss during the formation of the NCCL, estimated by subtracting (A) from (B) in *WearCompare*. Scale bar is in μm.

**FIGURE 4:**
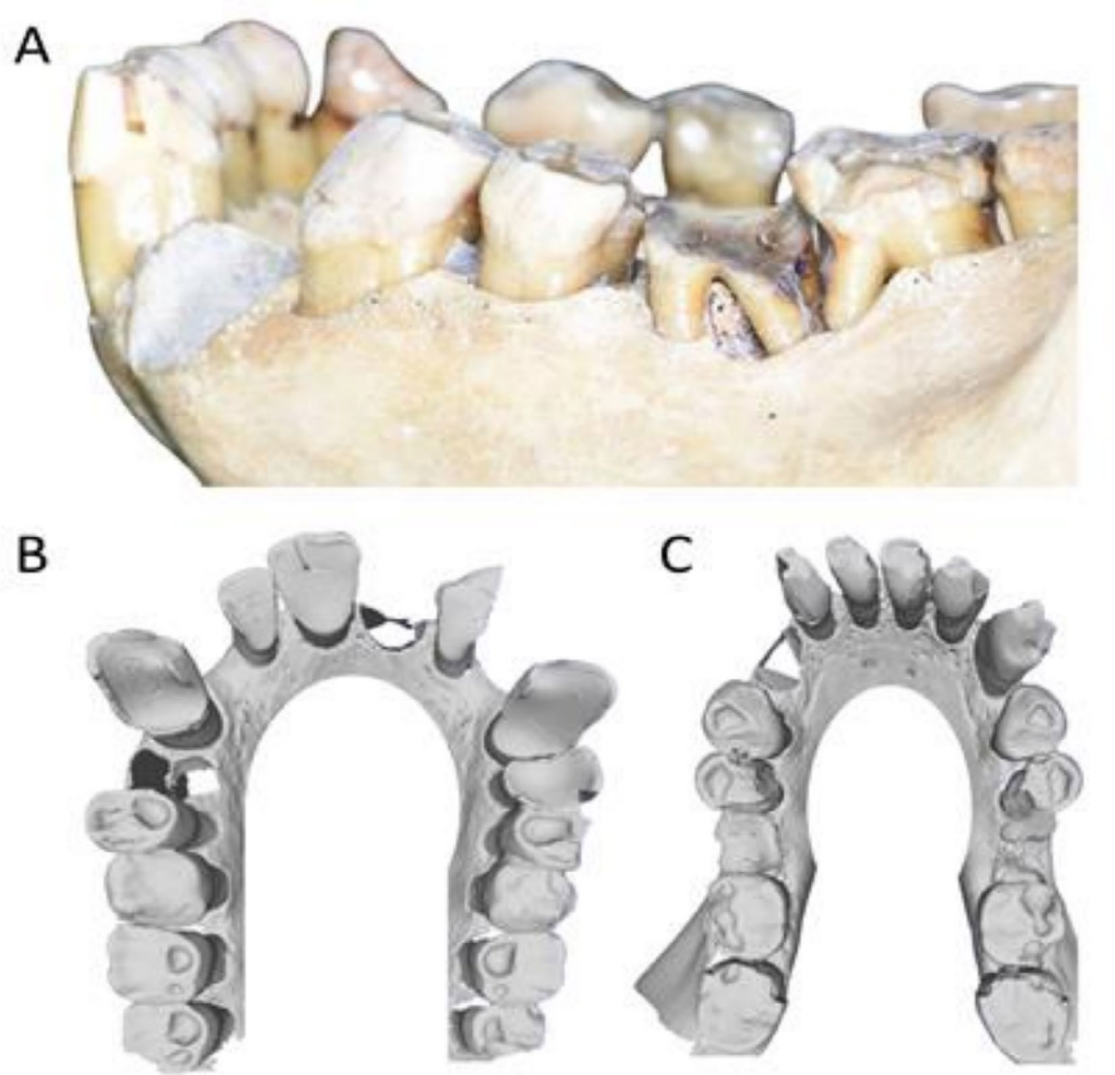
(A) buccal overview of the left mandibular dentition of FMNH 19026 ( *Pongo pygmaeus* ), showing periodontal disease in the region of the first and second molars. Occlusal overview of both the upper (B) and lower (C) dentitions. Both are 2D stills captured from 3D models created using an intraoral scanner (3Shape Trios 4).

The estimated hard tissue loss was 12.5 mm^3^, characterized by a shallow and uniform semi‐circular profile across its surface (Figure 3A–C). The lower right second molar also displayed a lesion on the mesial root surface, appearing carious with dark coloration and rough texture. Both lower first molars show significant tissue loss, with the left (adjacent to the second molar with the NCCL) showing extensive steep wear that closely followed the contour of the NCCL, likely contributing to its tapered shape. Severe localized periodontal disease was present in the region of the NCCL, particularly affecting the first and second molars, with more generalized periodontal disease observed throughout the dentition (Figure 4A–C). There was no evidence of malocclusion in this region or elsewhere in the dentition (Figure 4B,C).

**FIGURE 5:**
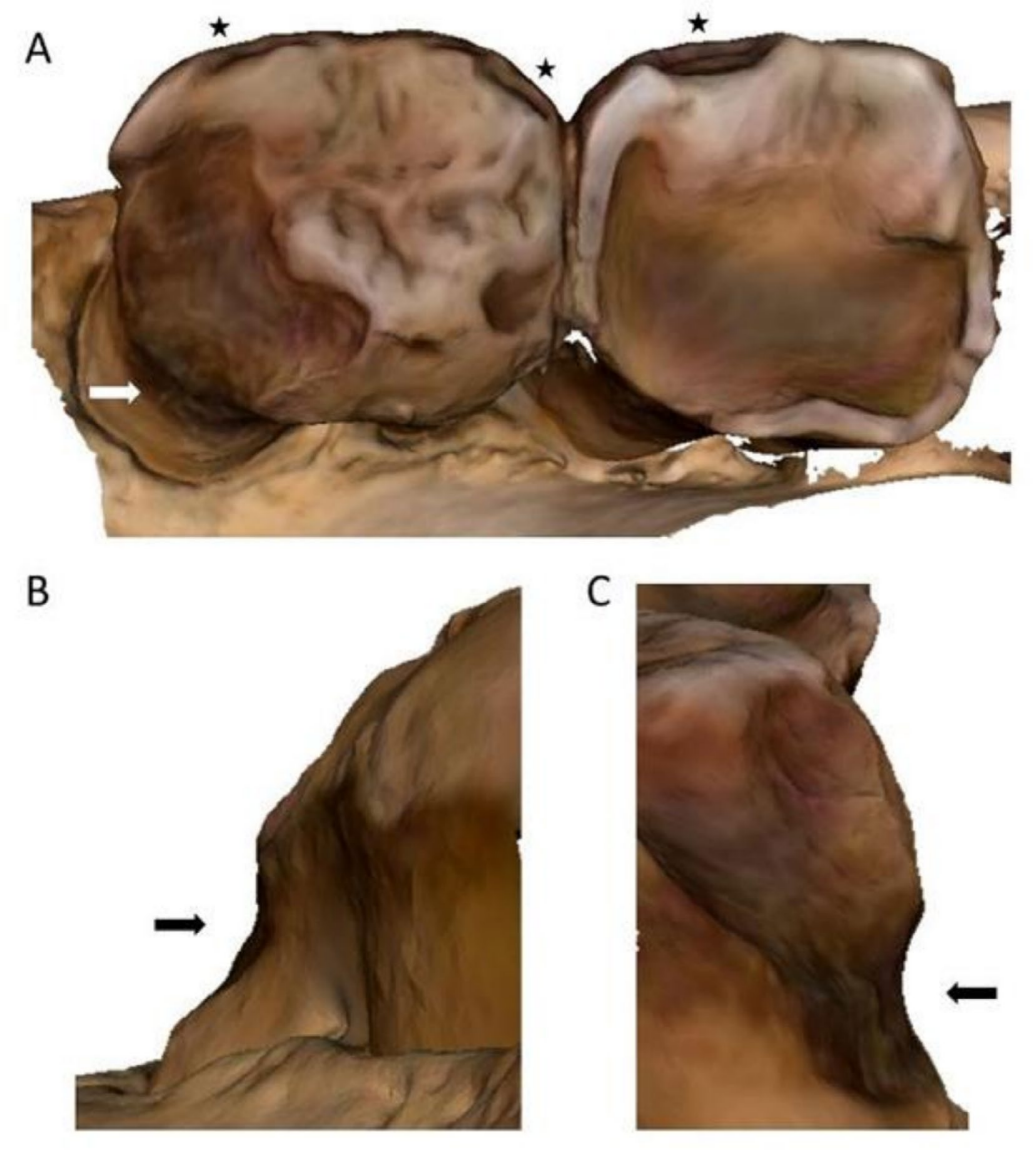
Specimen FMNH 33533 ( *Pongo pygmaeus* ), showing a lesion consistent with a NCCL on the distal aspect of the right third molar. (A) occlusal view with the NCCL on the distal surface (white arrow), with large occlusal antemortem fractures highlighted (black stars); (B) lingual view showing the tissue removed (black arrow); (C) buccal/distal view of the same lesion (black arrow). Each image is a still from a 3D PLY model created with a 3Shape Trios 4 intraoral scanner in high resolution mode.

Another orangutan specimen curated at the Field Museum (FMNH 33533; Figure 5) also displays an interproximal NCCL on the lower third molars and potentially second molars. The teeth had steep tooth wear, with large fractures on many teeth (Figure 5A). The NCCLs seem to be an extension of the occlusal wear. Once the enamel was worn away, the root surface likely became exposed, allowing attrition to continue. The concave surface of the groove justified its classification as an NCCL. The lesion was slightly darker than the surrounding root dentine (although similar to the dentine on the occlusal surface) and had a rough texture in some areas. Although caries cannot be excluded, an NCCL seems more likely based on shape and surface features. Due to the specimen condition, molding was not feasible.

**FIGURE 6:**
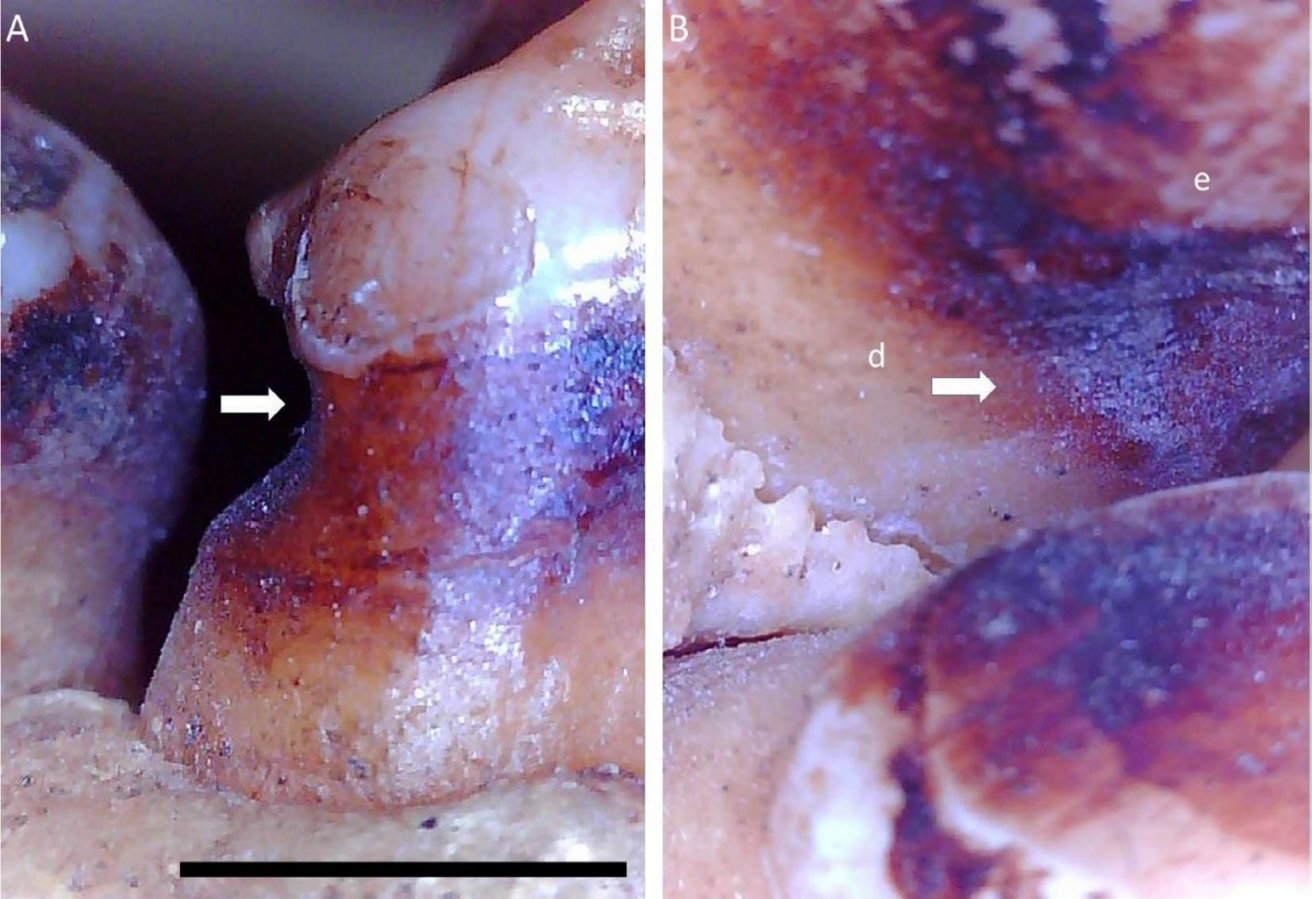
*Gorilla gorilla gorilla* specimen (PCM Z I 17). Lower left canine showing an interproximal groove (distal surface) consistent with NCCL: (A) lingual view; (B) close up of the NCCL, as seen from the buccal‐distal (d = dentine; e = enamel). Both images taken using a Dinolite microscopic digital camera. Scale bar 5 mm.

An interproximal NCCL similar to hominin toothpick grooves was observed on the distal surface of the lower left canine in a *Gorilla gorilla gorilla* specimen (PCM Z I 17; Figure 6). Close up Dino‐lite Images (Figure 6B) potentially indicate the presence of fine striations oriented buccal‐lingually, however more detailed analysis was not feasible. The dark coloration was likely associated with plaque and/or dietary items, which stained the root surface due to substantial gingival recession in this region (see Albrecht et al. 2024). The shallow U‐shaped groove did not have deep cavitation or a rough demineralized texture, supporting a non‐carious etiology. The tissue loss seems to follow the contours of the presumed gum line, and in places has a relatively smooth and shallow uniform appearance, suggesting erosion cannot be ruled out.

**FIGURE 7:**
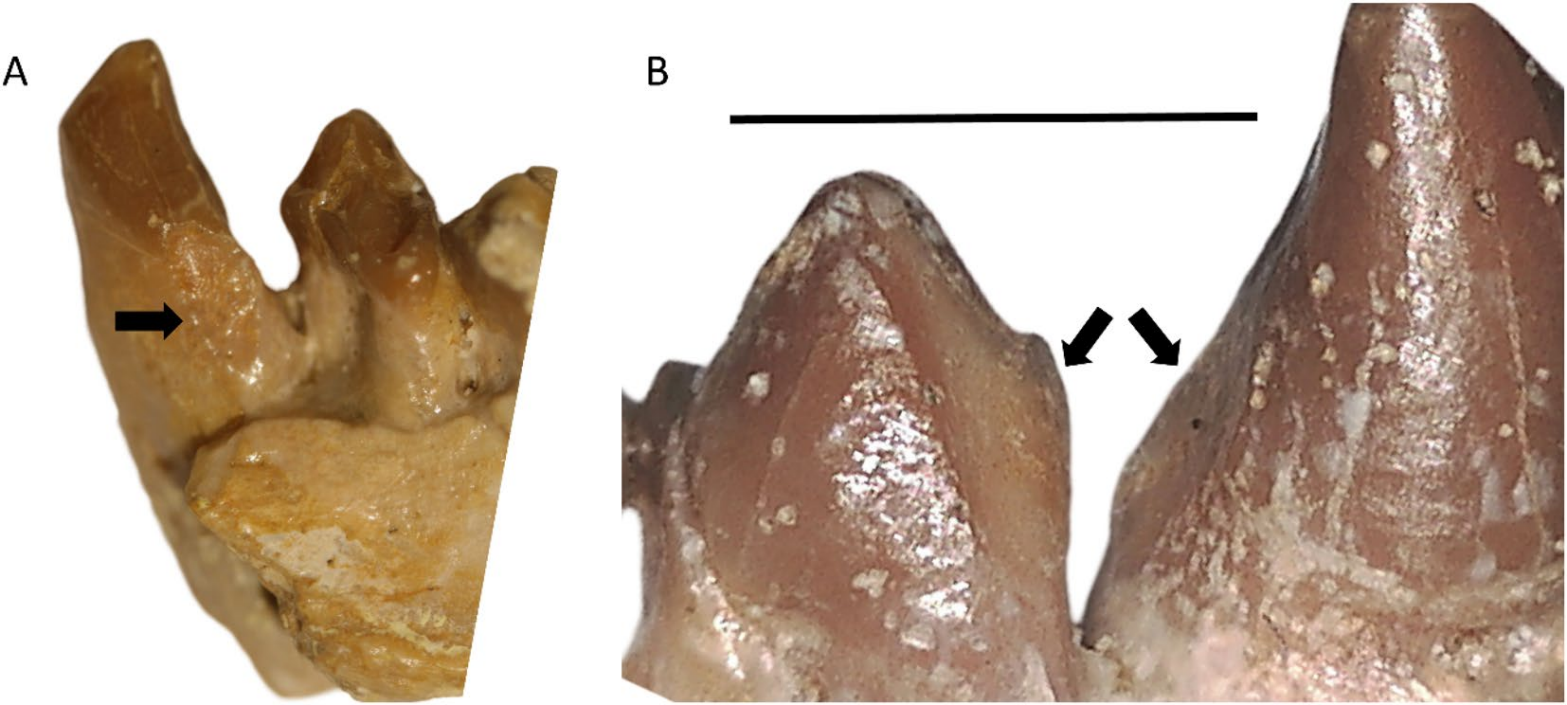
NCCL in an *Aegyptopithecus zeuxis* specimen (DPC 1042): (A) lingual view of the NCCL on the lower right canine (black arrow); (B) buccal view of the same NCCL (right black arrow) and corresponding wear on the mesial surface of the adjacent premolar (left black arrow). This wear on both teeth is created through contact with the upper canine, likely in a honing complex process. Scale bar 5 mm.

**FIGURE 8:**
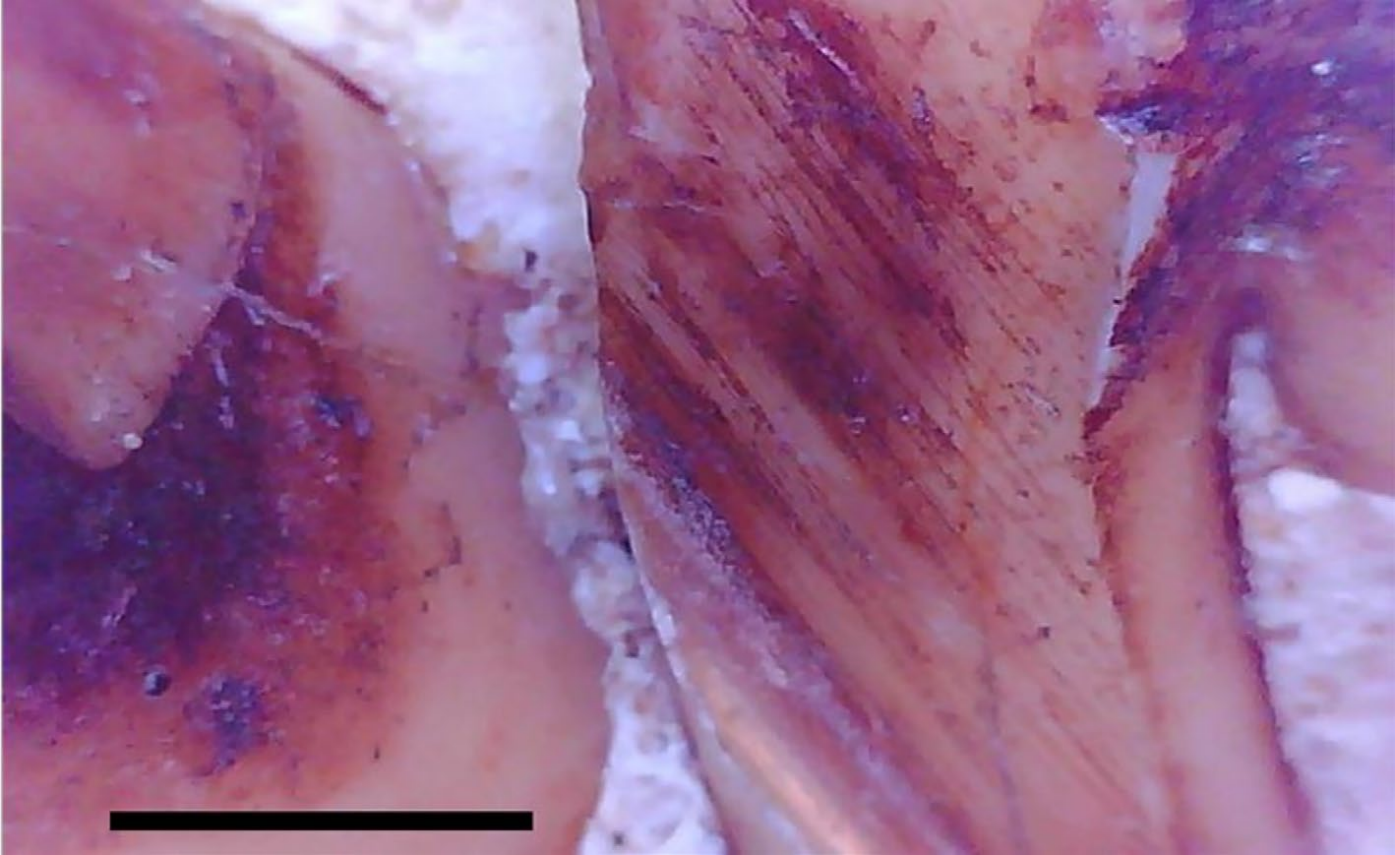
NCCL in a *Gorilla gorilla gorilla* specimen (PCM M 690, 2nd series), with the mesial root surface of the lower left third premolar extensively worn, caused by honing of the upper canine. Tooth crown towards the top of the image and apical of the root below. Scale bar 5 mm.

Two other specimens showing NCCL indicative of abrasion/attrition were teeth involved in the canine honing complex. This included the lingual surface of a lower canine in an early anthropoid specimen (DPC 1042, *Aegyptopithecus zeuxis*; Figure 7A), which is associated with substantial wear on the mesial surface of the mesial premolar (Figure 7B). Both of these wear facets were likely caused by contact with the upper canine in a honing capacity. Similarly, a *G. gorilla gorilla* specimen (PCM M 690, 2nd series) had an NCCL on the mesial root surface of the lower left third premolar, likely related to honing action of the upper canine (Figure 8). Given that this form of tissue loss may not represent a pathological process, its classification as an NCCL is further addressed in the Discussion. However, based on the inclusion criteria applied in this study, and the fact that in isolated fossil teeth such inferences may not be feasible, these lesions are reported here.

**FIGURE 9:**
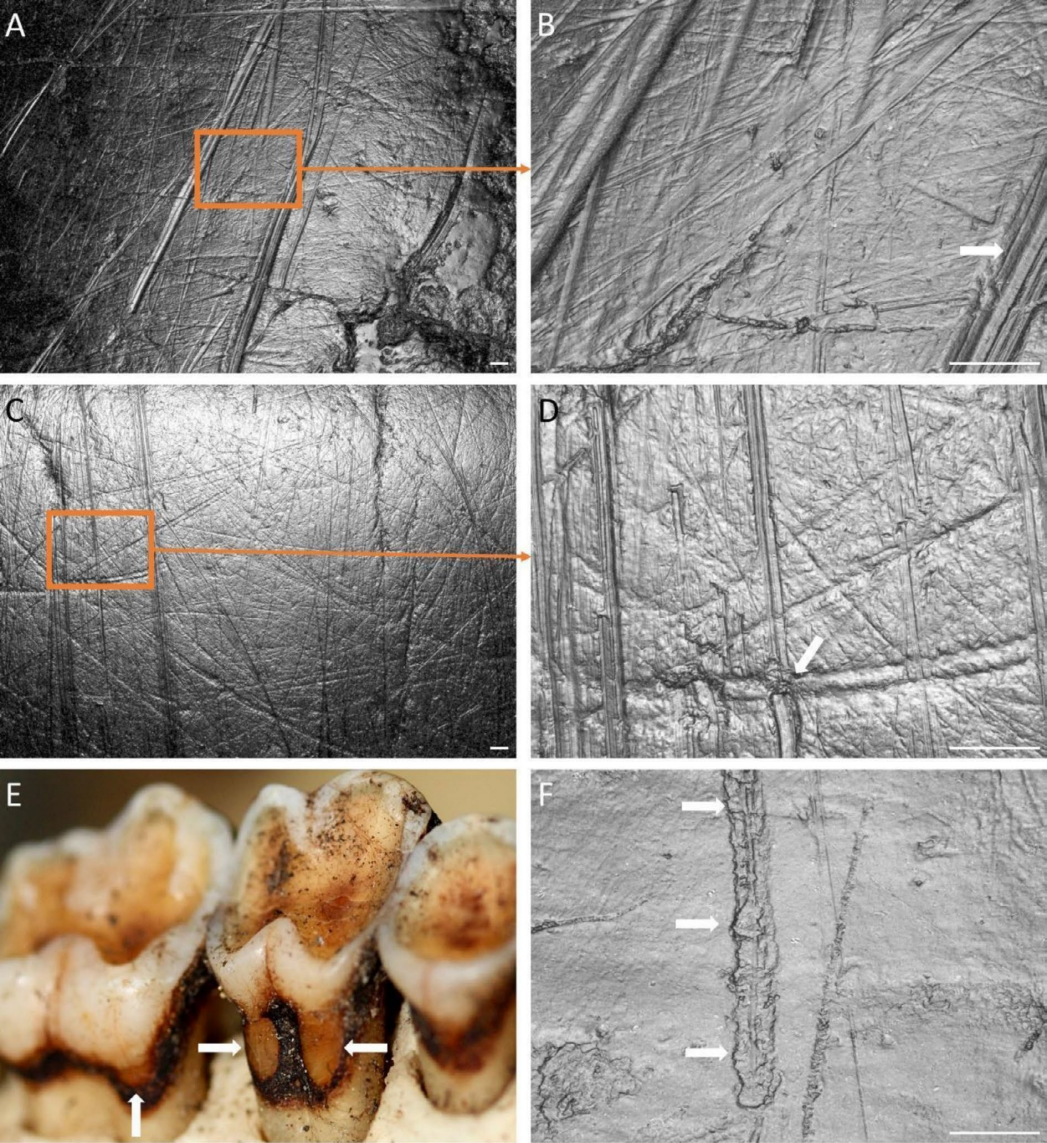
Japanese macaques ( *Macaca fuscata* ) from Koshima Island, Japan, showing NCCLs on the lingual root surface of upper molars: (A) PRI 7326, upper right third molar (20× zoom); (B) same tooth but 100× zoom (note parallel thinner striations within the large groove, white arrow); (C) PRI 7330, upper right first molar (20× zoom); (D) same tooth but at 100× zoom (note V‐shaped cross section and crossing of two striations, white arrow); (E) PRI 7388, upper right first molar (NCCL: White arrows); (F) PRI 7388, upper right first molar (100× zoom; white arrows: Hertzian cones). Scale bar 25 μm. For the microwear images, the right side of each image is distal, left side mesial, and top is occlusal.

In a population of Japanese macaques ( *Macaca fuscata* ) from Koshima Island, Japan, nine individuals (50% of the sample) had at least one NCCL. They show atypical wear elsewhere in the dentition, likely associated with accidental ingestion of sand (Towle, MacIntosh, et al. 2022). NCCLs were commonly found on the lingual root surface of upper molars (Figure 9). The NCCLs share the same characteristics, being shallow with a smooth surface; however, aligned striations of varying sizes (some > 25 μm) were visible microscopically (Figure 9). Other key microwear characteristics were identified, including parallel finer striations, either parallel or within the larger groove, Hertzian cones, and striations with V‐shaped cross‐sections.

**FIGURE 10:**
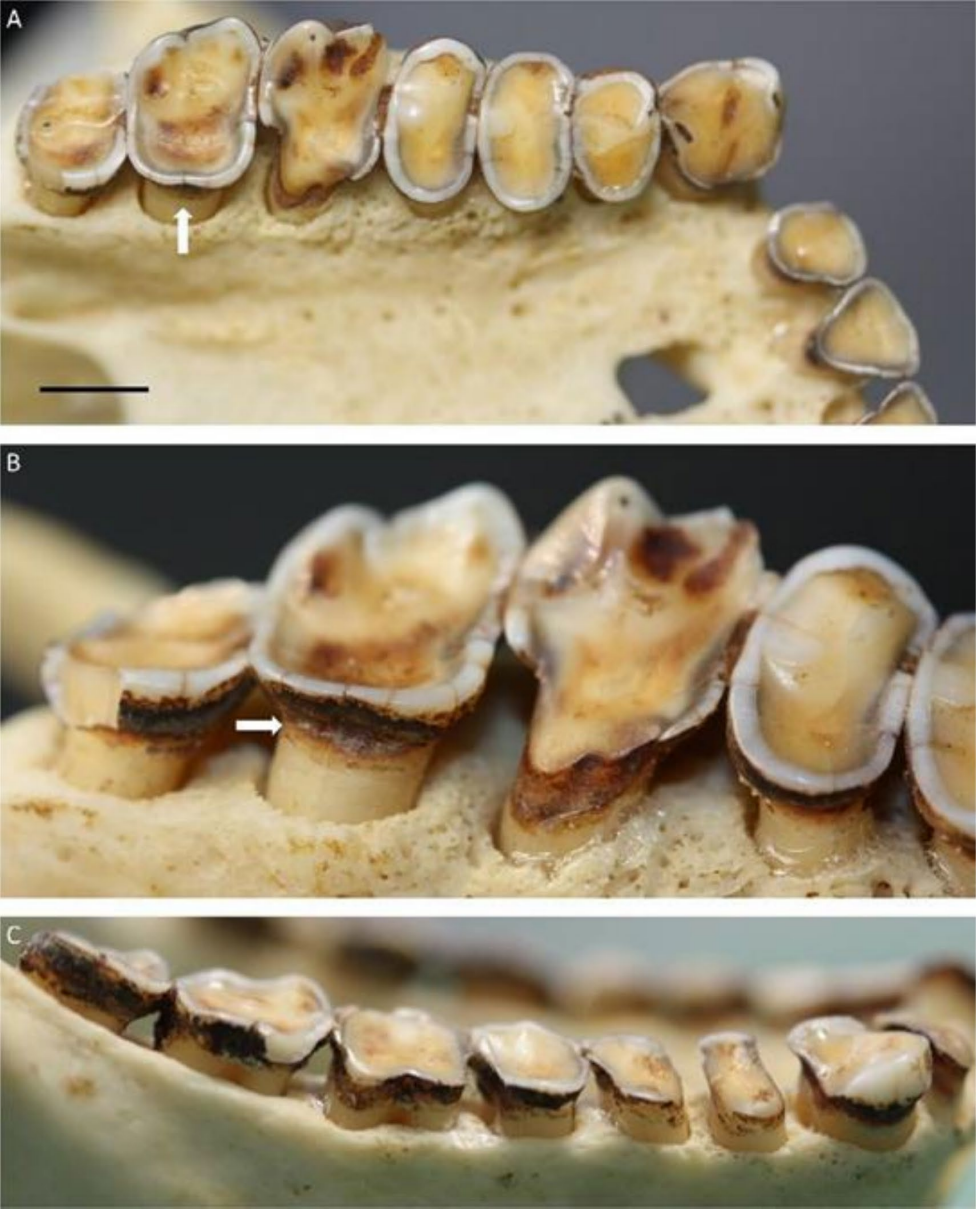
*Lagothrix lagothricha* specimen (PRI 683) with a NCCL on the lingual surface of the upper right second molar. (A) overview of the occlusal wear in the upper right dentition, with the NCCL highlighted (white arrow), the extreme wear on the adjacent first molar likely facilitated this root lesion to form; (B) close up of the NCCL (white arrow); (C) showing the extensive occlusal wear in the lower dentition of the same individual. Scale bar 5 mm.

A Brown woolly monkey specimen (PRI 683; *Lagothrix lagothricha* ) had a U‐shaped NCCL on the lingual surface of the upper right second molar (Figure 10). The lesion was associated with extreme wear on the dentition (Figure 10A,C); the adjacent first molar has complete enamel loss on the lingual distal corner of the occlusal surface, adding further support for a NCCL etiology. However, carious activity cannot be fully ruled out, due to the discolored film covering all exposure non‐occluding exposed surfaces (see Albrecht et al. 2024), making texture and microwear assessment unfeasible. The extreme beveled wear on anterior teeth and steep wear in some posterior teeth, as well as the NCCL itself, resembles wear features observed in the aforementioned Japanese macaque ( *Macaca fuscata* ) individuals and fossil *Homo* specimens.

### 3.2 Shallow, Smooth, NCCLs That Follow the Presumed Gumline Indicative of Erosion

**FIGURE 11:**
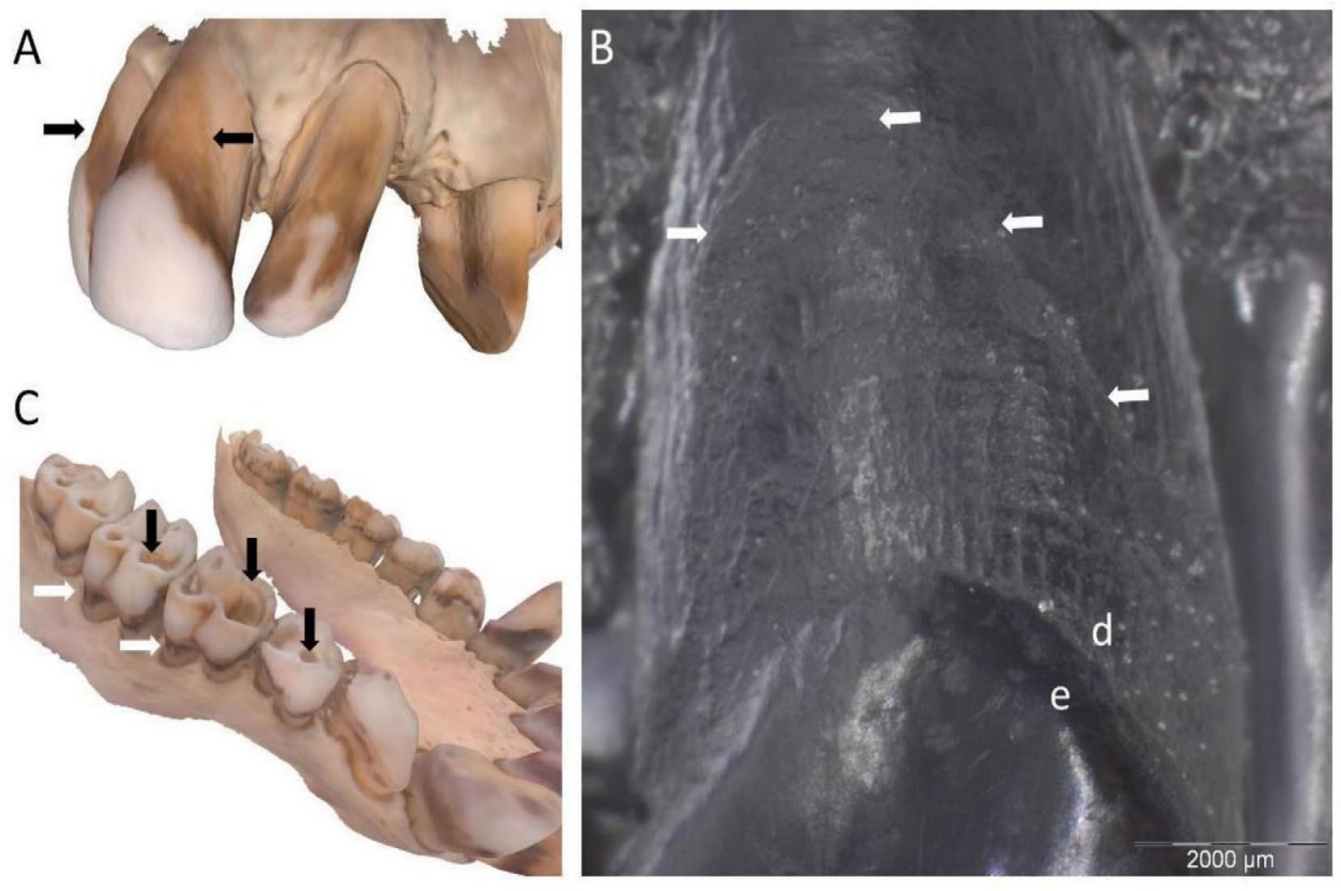
Non‐carious cervical lesions (NCCLs) in two *Lophocebus albigena johnstoni* individuals. (A) FMNH 27537: Central incisors with substantial gingival recession and root tissue loss that tapers following what was likely the gum‐line in life (black arrows); (B) Upper right central incisor showing microscopic detail of the NCCL in (A), note the tapered appearance of the lesion moving cervically (white arrows; e, enamel; d, dentine), and no obvious large striations as seen in examples of abrasion/attrition NCCL; (C) FMNH 24298: This individual shows very similar NCCL on the anterior teeth as well as the posterior teeth (white arrows), and is associated with deep occlusal cupping of the dentine, potentially relating to erosion wear (black arrows).

Several individuals exhibited root lesions suggesting a more complex etiology, with erosion a potential contributing factor in NCCL formation. Notably, two *Lophocebus albigena johnstoni* specimens (Figure 11) displayed pronounced incisor lesions, with tissue loss tapering cervically along the presumed gingival margin. Posterior teeth also showed NCCLs (Figure 11C), suggesting that all exposed roots (likely due to gingival recession) were affected. Macroscopically, these NCCLs appeared shiny and smooth; microwear analysis revealed a surface lacking distinct, large striations or any common orientation of smaller striations (Figure 11B). Additionally, these lesions are accompanied by other potential signs of erosion, such as deep cupped‐out dentine on posterior teeth (Figure 11C).

**FIGURE 12:**
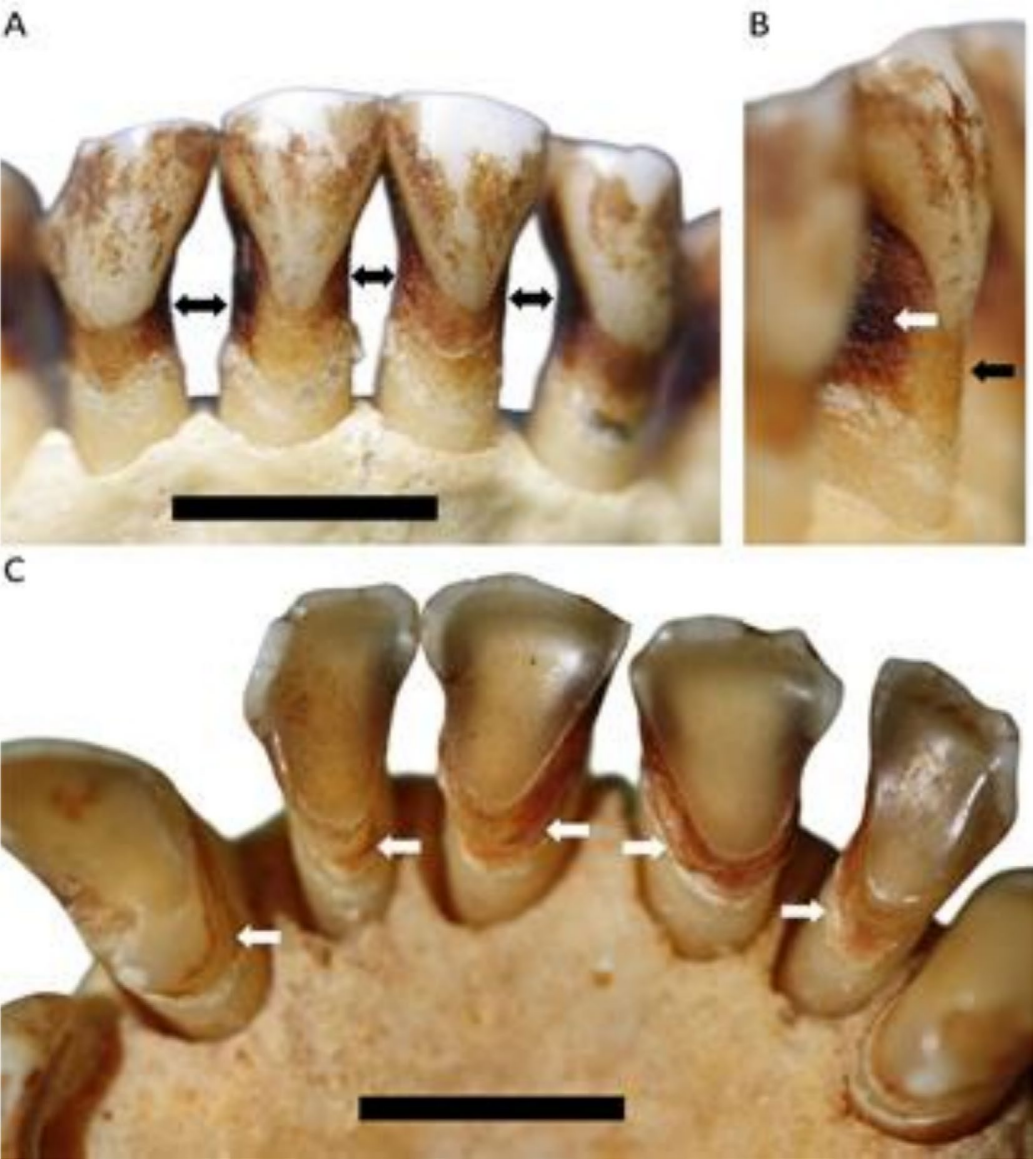
Potential non‐carious cervical lesions and/or dental caries: (A) Raffles' banded langur ( *Presbytis femoralis* ; PRI 4565); (B) Same individual showing close up of lower right central incisor, distal root surface. The root appears to show clear signs of a NCCLs, but deep within the interproximal areas is rough demineralized tissue suggesting carious activity (white arrow) activity, a (C) PRI 11579 ( *Cercopithecus denti* ), with substantial lingual wear, likely NCCLs related to erosion, but unusual caries lesions cannot be ruled out. Scale bars are 5 mm.

In several extant primates, distinguishing between NCCLs and root caries presents diagnostic challenges, especially when both conditions occur in similar locations. This complication is evident in species such as the Raffles' banded langur ( *Presbytis femoralis* ; PRI 4565, PRI 4557 and PRI 4559; Figure 12) and Dent's mona monkey ( *Cercopithecus denti* ; Figure 12), where individuals show analogous lesions to those found in Johnston's mangabey ( *Lophocebus albigena johnstoni* ), predominantly affecting the anterior dentition. In some cases, caries is found on adjacent or even in the same tooth, complicating the differentiation between NCCLs and caries (Figure 12B). Distinguishing NCCLs and caries in osteological and paleontological samples remain challenging due to overlapping morphological characteristics and lack of diagnostic tools available in clinical settings.

## 4 Discussion

The hypotheses in this study—(1) that erosion and abrasion‐related lesions are present in non‐human primates and reflect acidic fruit or hard object diets and (2) that abfraction and toothpick grooves are not present in non‐human primates—were only partially supported. While the overall prevalence of NCCLs in the anthropoid primates studied here was lower than in contemporary human populations, and no abfraction‐type lesions were identified, NCCLs resembling toothpick grooves in fossil *Homo* samples were identified in the samples analyzed here. This suggests toothpick‐type lesions are not exclusive to the genus *Homo* and may expand our understanding of the behaviors or processes that create these types of lesions.

Our findings also highlight the diverse presentations of NCCLs in non‐human primates, which can be categorized into two main types: (1) lesions associated with smooth and shallow tissue loss on all exposed root surfaces, indicative of erosive wear; (2) U‐shaped localized tissue loss (i.e., not affecting all exposed root surfaces), with fine parallel striations visible within the lesion, indicative of attrition and/or abrasion. These findings have implications for the study of fossil hominin and modern clinical samples.

### 4.1 Fossil Hominin Implications

The identification of interproximal NCCLs in three ape specimens (*Pongo* FMNH 19026 and FMNH 33533, *Gorilla gorilla gorilla* specimen PCM Z I 17), as well as similar lesions on other tooth surfaces in Koshima Island Japanese macaques ( *Macaca fuscata* ) and *Lagothrix lagothricha* specimen (PRI 683), provides evidence that NCCLs in non‐human primates share characteristics with those described in fossil *Homo* samples. In these examples, overall tooth wear patterns associated with toothpick grooves in *Homo* were also present, including substantial occlusal wear; beveled wear on the anterior teeth; fractures on the teeth with NCCLs or directly adjacent teeth; and associated pathologies such as severe periodontal disease and caries. Similar root tissue loss can be seen in published images of osteological ape specimens from other locations, suggesting these lesions are not restricted to particular populations (e.g., Albrecht et al. 2024), or to anthropoid primates (e.g., Figure 9 in Cuozzo and Sauther 2006).

The NCCL in *Pongo* specimen FMNH 19026 is perhaps the clearest example of a toothpick groove in this study. The presence of a carious lesion beneath the NCCL could be used to infer an object might have been repeatedly inserted into the affected region to alleviate pain or discomfort. While tooth‐picking and other tool‐use behaviors remain plausible explanations for atypical tooth wear in non‐human primates, supported by observations of non‐dietary items placed in the mouth (e.g., Watanabe et al. 2007; Leca et al. 2010), and documented instances of captive orangutans using their teeth for tool manipulation (O'Malley and McGrew 2000), analysis of the full dentition of this orangutan suggests a tooth‐picking origin is unlikely. The NCCL is associated with severe tissue loss on the adjacent first molars, with its tapered shape aligning with the outline of the first molars' steep wear profile. Additionally, the carious lesion and periodontal disease affecting the region also appear linked to tissue loss on the neighboring first molar.

The hypothesis that behaviors such as stripping vegetation could contribute to the formation of unusual wear patterns warrants further investigation. Larger orangutans have been observed opening *Neesia* fruits by holding them in the mouth and pulling using multiple appendages (Van Schaik and Knott 2001). They have also been documented making wooden tools, a process that involves stripping bark off twigs with their teeth, as well as holding the completed tool in the mouth while performing different functions (Van Schaik et al. 1996). While such behavior might contribute to localized wear such as the one found in FMNH 19026 (i.e., extreme steep tissue loss on first molars and root wear on the adjacent second molars), a masticatory origin is also plausible. Orangutans consume a variety of fruits, with seasonal shifts in their diet. During periods of low fruit availability, they rely on tougher fallback foods such as bark, pith, and unripe seeds (Galdikas 1988; Knott 1998; Vogel et al. 2015). The abrasive properties of these foods, especially when combined with potential ingestion of environmental grit, can lead to significant dental tissue loss, often resulting in rapid dentine exposure (Morrogh‐Bernard et al. 2009; Fiorenza et al. 2024). The abrasion of food particles and environmental grit across the root could account for the macro and microscopic features of the NCCL observed in this individual. In this scenario, fine buccal‐lingual striations within the NCCL reflect the passage of food and/or grit across the exposed dentine surface, influenced by chewing dynamics, saliva flow, and swallowing.

In fossil hominins, NCCLs are similarly associated with substantial tissue loss on adjacent teeth, resembling the NCCL in the FMNH 19026 orangutan specimen (e.g., Eckhardt and Piermarini 1988; Durband et al. 2012; Lozano et al. 2013). Some hominins, NCCLs are also associated with oral pathologies (e.g., Frayer et al. 2017; Fong 1991; Martinón‐Torres et al. 2011). Other examples of NCCLs in hominins are linked to heavy or steep tooth wear, either on the affected tooth or those adjacent (Ubelaker et al. 1969; Puech and Cianfarani 1988; Lalueza et al. 1993; Bermudez de Castro et al. 1997; Gracia‐Téllez et al. 2013; Lozano et al. 2013). NCCLs are also often associated with large interproximal wear facets and other interproximal features such as vertical grooving within the facets, complete removal of enamel above the interproximal contacts, and fractures (Berryman et al. 1979; Skinner and Sperber 1982; Turner 1988; Puech and Cianfarani 1988; Urbanowski et al. 2010; Sun et al. 2014; Nowaczewska et al. 2021). *Homo* specimens seem particularly prone to these interproximal features (Villa and Giacobini 1995; Estalrrich et al. 2011; Willman 2016; Towle et al. 2017; Belcastro et al. 2018). Similarly, heavy interproximal wear is often associated with periodontal disease or continuous eruption, which can expose the root to the oral environment and render interproximal surfaces susceptible to undercutting forces in posterior teeth (see fig. 1 of Berryman et al. 1979; fig. 1 of Turner and Cacciatore 1998; fig. 2 of Margvelashvili et al. 2013). Lastly, finite element modeling has identified high stress concentrations near the CEJ, where toothpick grooves commonly form (Macho and Spears 1999; Macho et al. 2005; Benazzi et al. 2014; Najafzadeh et al. 2024).

Therefore, despite the prominence of the toothpick hypothesis, caution is necessary when attributing all hominin NCCLs to such behaviors; other causes or etiologies should be considered within or between populations, especially given the complex forces acting across the dentition (Molnar and Ward 1977; Patterson and Kingsnorth 1984; Towle, MacIntosh, et al. 2022). Additionally, the absence of similar NCCLs in recent human samples, despite known toothpick use, suggests that this hypothesis may not be universally applicable (Weidenreich 1937; Wallace 1974; Brown 1991; Ungar et al. 2001). Other hypotheses should be investigated, such as Wallace's (1974), who suggested that grit might pass through interproximal regions during mastication. A potential counterargument to the grit‐related etiology is that NCCLs would also be expected to feature atypical wear on the other crown and root surfaces (Molnar 2011). Koshima Island macaques consuming sand‐laden food present large scratches on their anterior teeth and beveled incisors, along with NCCLs on posterior teeth (Towle, MacIntosh, et al. 2022), similar to some fossil hominins (e.g., Bonfiglioli et al. 2004; Clement 2008; Frayer et al. 2017). NCCLs are not restricted to interproximal surfaces in archaeological and fossil *Homo* samples. Detailed studies of NCCL variation have revealed a range of features, including affected tooth surfaces, shapes, and sizes (e.g., Ubelaker et al. 1969; Frayer and Russell 1987; Lukacs and Pastor 1988; Turner and Cacciatore 1998; Ritter et al. 2009). Recent advancements in the clinical assessment of NCCLs, experimental work on lesion development, and the presence of similar lesions in non‐human primates underscore the importance of reevaluating these features in fossil hominins.

For example, microwear features within NCCLs of Japanese macaques from Koshima Island are consistent with those often linked to tool‐use behaviors in *Homo* samples (e.g., large striations, > 20 μm; presence of parallel, finer striations, either parallel to or within a larger groove; Hertzian cones; striations with V‐shaped cross‐sections; de Castro et al. 1988; Lozano‐Ruiz et al. 2004; Estalrrich and Rosas 2013). As the microwear patterns in these macaques are caused by sand ingestion (Towle, MacIntosh, et al. 2022), further investigation is needed into the broader range of tooth wear patterns, especially those commonly interpreted as tool‐use behavior in fossil *Homo* individuals. This re‐evaluation could provide valuable insights into the behavioral and ecological contexts of tooth wear across species.

### 4.2 Dental Erosion and Clinical Implications

The potential erosive tooth wear observed in *Lophocebus albigena* aligns with dietary studies on members of the gray‐cheeked mangabey complex, which preferentially consume ripe fruit and pulp while avoiding less ripe alternatives (Masette et al. 2015). Similar dietary habits are seen in other species exhibiting these features ( *Cercopithecus denti* and *Presbytis femoralis* ), which also primarily eat ripe fruits (Davies et al. 1988; Olaleru 2017). However, these species also eat a wide variety of other foods. Therefore, detailed field studies on individuals or populations showing NCCLs are required to better understand foods and processing behaviors leading to the formation of these lesions. Root surfaces exposed by gingival recession or periodontal disease are particularly vulnerable to wear (Igarashi et al. 2017; Goodacre et al. 2023), and we suggest the acidity of the diet is the likely cause of hard tissue loss. In particular, the softer dentin erodes, resulting in wide, shallow, and smooth lesions affecting anterior teeth responsible for the initial processing of these fruits. However, further research on living primates is needed to test this hypothesis and to confirm the involvement, or significance, of acid erosion in the development of NCCLs.

Our findings also highlight challenges in determining the cause of some root lesions. In particular, distinguishing between carious lesions and NCCLs can be difficult in osteological and paleontological contexts where soft tissue is absent and taphonomic agents can change surface coloration and texture. Additionally, in these settings, changes in mineral concentration of the underlying dental tissue are commonly not reliable. This ambiguity may also stem from the multifactorial nature of many root lesions, with similar etiologies potentially yielding lesions with similar presentations. Species such as *P. femoralis* and *C. denti* , which display signs of erosive wear NCCLs, also show caries in the same regions in other individuals; even the same tooth can show potential signs of both types of lesions (e.g., Figure 12B). Similar lesions have been described in clinical human samples (e.g., Igarashi et al. 2017, their fig. 3b). While some lesions are clearly carious, with deep cavitation and substantial demineralization, and are thus excluded from our NCCL classification (see Towle, Irish, et al. 2022 for examples), it is possible that early‐stage caries could be misidentified as erosion. Further research differentiating these processes is warranted and could help clarify if root lesions described in early anthropoids (e.g., *Propliopithecus*) are best classified as caries (Towle, Borths, and Loch 2024) or NCCLs.

The absence of abfraction lesions in the present study is not related to a lack of abrasive materials, as seen by the NCCLs described and the high rates of occlusal tooth wear observed in wild primates. Similarly, primates that commonly consume erosive fruits also do not show abfraction lesions. Together with the lack of abfraction examples in archaeological and fossil *Homo* samples, this suggests that abfraction‐related NCCLs documented in contemporary clinical settings may not be solely caused by stresses and micro‐fractures forming in these regions. Our results therefore support the hypothesis that a combination of erosion and abrasion related to current human behaviors and diets (e.g., tooth brushing and carbonated/acidic drinks) is responsible for the high prevalence of abfraction lesions. This is supported by recent experimental evidence (Denucci, Towle, et al. 2024; Denucci, Stone, and Hara 2024).

The prevalence of NCCLs is lower in wild non‐human primates compared to humans today, where up to half the population can be affected, with a substantial increase by age cohort (e.g., Teixeira et al. 2020; Kitasako et al. 2021). This supports the idea that unique human behaviors and diets contribute to a higher prevalence of NCCLs in clinical settings, though studies considering the effects of aging are needed to further test this hypothesis. The frequency of NCCLs in archaeological and fossil contexts is less clear, and comparisons are difficult due to the fragmentary nature of these collections. A study on NCCLs in archaeological samples from North America and Europe by Ritter et al. (2009) found a low prevalence, with three of the five samples showing no NCCLs, and a total prevalence of 6.7%. Around a quarter of the adult Krapina Neandertals showed toothpick‐type grooves (Frayer and Russell 1987), with comparative samples showing lower prevalence ranging from 2% to 8.1%. Therefore, at present, there is little evidence to suggest that *Homo* (with the exception of contemporary modern populations) has a higher prevalence of NCCLs compared to other primates.

### 4.3 Terminology, Future Research, and Limitations

Differences in tooth morphology among primates complicate the terminology surrounding NCCLs, particularly when discussing “lesions” across species. NCCLs associated with the canine‐honing process may not be classified as such in future research. In many cases, such wear is unlikely to be pathological and may represent normal physiological wear, whether on the canine or adjacent premolar. However, this interpretation will depend on the species. Similarly, many catarrhine primates have evolved to lose most of the enamel on the lingual surface of lower incisors, leaving dentine exposed shortly after eruption (Kupczik and Lev‐Tov Chattah 2014), and affecting how wear in these regions is described. This may be beneficial for comparative studies, deepening our understanding of species‐specific traits.

Specimen DPC 1042 (*Aegyptopithecus zeuxis*) has been identified as female due to its small dentition (Fleagle et al. 1980; Kay et al. 1981). In earlier studies, significant wear on the mesial margin of the mesial premolar has been highlighted in this specimen and observed in other *A. zeuxis* specimens. These teeth likely form a canine‐honing complex, like that in many modern anthropoids, which serves to maintain the sharpness of the upper canine over time (Zingeser 1969; Swindler 2002; Delezene 2015). This large NCCL, likely accommodating a large upper canine, when considered alongside wear on the adjacent third premolar, could provide valuable additional insights into the evolution of this part of the dentition in anthropoid primates. Future research drawing on larger comparative samples from diverse living anthropoid taxa will be essential to determine how consistently this form of root wear occurs within and between populations and between males and females, associated with the canine‐honing complex.

The main limitation of this study is the relatively small sample size for several taxa. Here, half of the extant primate species are represented by fewer than 10 individuals. While this study provides broad taxonomic coverage, small sample sizes are unlikely to reflect the true average prevalence of NCCLs within each taxon. Reliable prevalence estimates for a relatively infrequent pathology such as NCCLs will require substantially larger samples (e.g., 50+ individuals per species). Obtaining such data should be feasible for many groups, as many museum collections house large numbers of specimens for particular taxa. The present study should therefore be viewed as an initial exploration of this topic, demonstrating that NCCLs occur across a range of primates, and lesion presentations may be comparable to those described for fossil hominins. Future research on larger comparative samples will be essential to identify prevalence and frequency of occurrence among taxa.

A further limitation of the present study is the lack of comprehensive comparative data on root lesions more generally in primate osteological and fossil collections. While caries and NCCLs have received considerable attention in both clinical and archaeological research, both remain underreported in non‐human primates. This gap makes it difficult to evaluate their morphological variability and etiology across different environmental or behavioral contexts. Systematic surveys of root lesions in large museum collections would not only improve prevalence estimates but also provide a stronger basis for distinguishing different pathological, behavioral, and taphonomic factors. Our results suggest multiple pathologies in the same tooth position may be common (i.e., caries and NCCLs). This complexity is also evident in fossil hominins, where toothpicking has been suggested as a response to pain or discomfort caused by carious lesions (and hence why both lesions would be present on the same tooth). Detailed analyses using techniques such as Micro‐CT scanning (to evaluate changes in mineral density) or histology (to investigate structural changes and lesion progression) are recommended to elucidate the interplay between these different pathologies.

## 5 Conclusion

This study provides prevalence data and descriptions of NCCLs in both extant and extinct anthropoid primates, allowing future comparisons with clinical and fossil *Homo* lesions. Our findings suggest that the formation of NCCLs can be influenced by a range of factors, including masticatory patterns, dietary abrasiveness and acidity, and underlying periodontal disease. No abfraction‐like lesions, which are common in human clinical settings, were observed in this sample, supporting the hypothesis that abfraction is associated with behaviors and dietary habits of contemporary humans. Furthermore, some NCCLs resembled the so‐called “toothpick grooves” in fossil hominins, suggesting that these features may result from normal mastication and food processing behaviors rather than oral hygiene‐related tool use. These findings highlight the need to re‐examine fossil hominin samples. Future comparative studies of non‐human primates, including direct field observations, are essential for refining our interpretations of NCCLs in archaeological and paleontological contexts. There might also be implications in clinical settings, improving our understanding of the behavioral and physiological factors underlying the formation of NCCLs.

## Author Contributions

**Ian Towle:** conceptualization, investigation, writing – original draft, methodology, visualization, writing – review and editing, software, formal analysis, project administration, data curation, funding acquisition. **Kristin L. Krueger:** investigation, writing – original draft, methodology, writing – review and editing, resources, visualization, data curation. **Kazuha Hirata:** investigation, writing – review and editing, visualization, methodology, formal analysis, data curation, resources. **Mugino O. Kubo:** investigation, methodology, visualization, writing – review and editing, formal analysis, data curation, resources. **Anderson T. Hara:** investigation, funding acquisition, writing – review and editing, resources. **Joel D. Irish:** investigation, funding acquisition, writing – review and editing, formal analysis, supervision. **Carolina Loch:** supervision, formal analysis, writing – review and editing, funding acquisition, investigation. **Matthew R. Borths:** investigation, writing – review and editing, visualization, resources. **Luca Fiorenza:** funding acquisition, investigation, conceptualization, visualization, methodology, formal analysis, supervision.

## Conflicts of Interest

The authors declare no conflicts of interest.

## Supporting information


**Data S1:** ajpa70132‐sup‐0001‐DataS1.xlsx.

## Data Availability

The data that supports the findings of this study are available in the Supporting Information of this article.
